# The Effect of Victory and Defeat on the Correlations of Stress Parameters Between the Horse and Rider in Kök‐Börü Equestrian Teams

**DOI:** 10.1002/vms3.70356

**Published:** 2025-04-24

**Authors:** Ali Rişvanli, İsmail Şen, Kanat Canuzakov, Askarbek Tulobayev, Abuzer Taş, Ruslan Salykov, Nezahat Ceylan, Ünal Türkçapar, Ulanbek Alimov, Arina Kazakbayeva, Ayday Cunuşova, Nur Abdimnap Uulu, Burak Fatih Yuksel, Mert Turanli, Muhammed Uz, Metin Bayraktar, Nuriddin Ruzikulov

**Affiliations:** ^1^ Faculty of Veterinary Medicine Kyrgyz‐Turkish Manas University Bishkek Kyrgyzstan; ^2^ Department of Obstetrics and Gynaecology Faculty of Veterinary Medicine Fırat University Elazig Türkiye; ^3^ Faculty of Sports Science Kyrgyz‐Turkish Manas University Bishkek Kyrgyzstan; ^4^ Department of Surgery Faculty of Veterinary Medicine Yuzuncu Yil University Van Türkiye; ^5^ Department of History Faculty of Humanities Kyrgyz‐Turkish Manas University Bishkek Kyrgyzstan; ^6^ Department of History Faculty of Humanities Ataturk University Erzurum Türkiye; ^7^ Faculty of Sports Science Kahramanmaras Sutcu Imam University Kahramanmaras Türkiye; ^8^ Faculty of Tourism Kyrgyz‐Turkish Manas University Bishkek Kyrgyzstan; ^9^ Department of Zootechny Faculty of Veterinary Medicine Fırat University Elazig Türkiye; ^10^ Samarkand State University of Veterinary Medicine Livestock and Biotechnology Samarkand Uzbekistan

**Keywords:** horse, Kök‐Börü, metabolism, physiology, rider, stress

## Abstract

The presented study outlines a research plan aimed at determining the effects of winning and losing situations on the relationship levels between the rider and horse's stress, metabolic, and physiological parameters in Kök‐Börü, a traditional equestrian team game. For this purpose, blood samples were collected from both the horses and riders of four different teams participating in two different Kök‐Börü games before and after the games. Cortisol, ACTH, beta‐endorphin, epinephrine, norepinephrine, T3 and T4 analyses were performed on the collected blood samples using species‐specific commercial ELISA kits. Additionally, biochemical and haematological parameters in the same blood samples were tested using an autoanalyser. Based on the obtained data, it was found that there were both positive and negative correlations between most biochemical and haematological parameters of the winning teams' horses and riders before and after the game. However, when examining the correlations between the hormonal parameters of the winning teams' horses and riders before the game, only a negative correlation was found between ACTH and T4 (‐0.529, p < 0.05), and no positive correlation was identified among any hormonal parameters. In conclusion, it was interpreted that in the equestrian team sport of Kök‐Börü, there are significant changes in the hormonal parameters, especially before and after the game, between the horses and riders of the losing teams. Furthermore, it was concluded that winning and losing situations in Kök‐Börü games did not have a significant impact on the correlations between haematological and biochemical parameters before and after the game for both the horses and riders.

## Graphical abstract

Kök‐Börü, a favoured traditional equestrian sport in Asia countries.

The relationship between riders and horses.

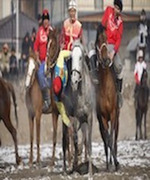

## 1 Introduction

Equestrian games and sports, traditionally a vital part of Central Asian culture, enjoy significant global appreciation. Prominent among these are mounted games such as Cirit, Polo and Kök‐Börü. Kök‐Börü, a favoured traditional equestrian sport, boasts super leagues and various lower league divisions, particularly in countries like Turkmenistan, Kyrgyzstan and Kazakhstan. This game is played on large, flat fields and is a competition between two teams, each with five, ten or fifteen players (Yücel, 2010; Gül et al. 2018). The game essentially involves players earning points by throwing dried, cut pieces of lamb or goat skin into the opponent's goal. The team accruing the most points within a set time is declared the winner (Kafkasyalı 2005; Kurt et al., 2017). While variations exist across countries, this core gameplay remains the same.

The historical relationship between humans and horses initially centred around the need for meat. However, over time, horses became primarily instrumental in transport. Today, horses are commonly used for speed, carrying loads, agricultural purposes, food, entertainment, sports and therapeutic riding programmes (Anderson et al. 1999; Edenburg 1999). Most studies analyse how past interactions shape animals’ perceptions of humans, whether positive, negative or neutral (Waiblinger et al. 2006). To gauge the impact of experiences, represented by the type and length of interactions, we need measurements and assessments. These include observations of behavioural and physiological tests (Hausberger et al. 2008).

The performance of both rider and horse can be influenced by their relationship. More research is crucial to understand safer and better methods of interacting with horses, explore different techniques that can promote positive bonding, and assess the impact of human management and care on equine welfare. Identifying and understanding behavioural cues from horses can minimise accidents and enhance performance (Jaeggin et al. 2005; Waran and Casey 2005). To ensure safe horse management, regular tracking of observations and physiological parameters is indispensable (Hausberger et al. 2008).

The existing literature on the stress‐related physiological responses between horses and riders is limited, primarily focusing on individual performances rather than team equestrian sports. These studies often analyse the emotional, psychological, behavioural, stress and physiological adaptations of both entities in racing, show, or therapy scenarios (König et al. 2017). It has been established that direct human‐horse interaction significantly influences a horse's emotional arousal; horses are notably sensitive to human presence and behaviour, including body language used by trainers (Fureix et al. 2009; Keeling et al. 2009). Such interaction can expose horses to potentially stressful situations like veterinary examinations, social isolation, physical and mental training and participation in equestrian competitions (Desmecht et al. 1996; Cayado et al. 2006; Schmidt et al. 2010a; Strzelec. et al. 2011; Kędzierski et al. 2012).

Stressful situations can lead to behavioural alterations and heightened emotional arousal in horses, negatively affecting performance, welfare and human safety. A horse's stress response relies on various factors, such as the stress factors’ scope and intensity, its past experiences, physiological condition and the constraints involved (Etim et al. 2013). Measurable stress reactions can be both physiological and behavioural, with various parameters as indicators of an animal's discomfort. An animal's perception of stress influences the intensity of hormonal and behavioural responses (Keeling et al. 2009; Merkies et al. 2014).

Among the physiological parameters used to assess body adaptation responses to stressful conditions, many haematological, biochemical and hormonal parameters have been widely studied in both humans and animals. These include epinephrine, norepinephrine, cortisol, ACTH and beta endorphins (Manteca 1998; Hada et al. 2003; Kyrou and Tsigos 2007; Risvanli et al., 2024; Risvanli et al., 2025). In healthy horses, cortisol concentrations may vary during the day and season and according to body condition score, whereas it is suggested that sex and age have no effect on cortisol levels (Aurich et al. 2015; Cordero et al. 2012; Hart et al. 2016). Cortisol reaches peak values in 10 to 20 min depending on time with a circadian rhythm (Lay et al. 1992). In horses, betaendorphin release into the blood is especially evident during stress reactions (Fazio et al. 2013). It is assumed that the release of opioids into the blood occurs at most 1 h after the occurrence of the stressor. It is suggested that betaendorphin modulates the activity of the hypothalamic‐pituitary‐adrenal axis and thus regulates ACTH secretion. It is stated that betaendorphin release from the pituitary gland in horses is synchronised with the initial phase of the stress reaction and thus may alleviate the negative effects of cortisol on the organism (Golynski et al. 2011). In horses, cortisol levels offer a potential indicator of stress, not just during equestrian competitions (Peeters et al. 2010; Strzelec. et al. 2011; Peeters et al. 2013), but also during training and transport (Schmidt et al. 2010a; Schmidt et al. 2010b). However, it is noteworthy that cortisol release and the activity of the hypothalamus‐pituitary‐adrenal axis rise not only due to stress but also due to physical activity (Desmecht et al. 1996). In a study (Risvanli et al., 2024), it was shown that winning or losing in Kök Börü games did not significantly affect the haematological or biochemical parameters of horses. However, horses from the winning teams have been reported to have exhibited cortisol, ACTH and beta‐endorphin levels and better stress management was observed.

In this study, we aim to understand how winning and losing situations in Kök‐Börü, a traditional equestrian sport, impact the stress, metabolic and physiological parameters of both the rider and horse.

## 2 Material and Methods

In this study, horses from four teams in the xxx Kök‐Börü Super League, which competed at xxxxx University's Ethnosport Facilities, were used as research subjects. Approval for the project was obtained from xxxxx University Experimental Animals Ethics Committee (document date and number: 20.01.2023‐ 2023/01) and xxxx Ministry of Health Human Experiments Ethics Committee (document date and number: 15.01.2023‐ 2023/03).

Our project included 40 clinically healthy stallions from the Kyrgyz breed. The health conditions of both horses (temperature, pulse and general body condition) and riders (parameters such as blood pressure, temperature and pulse were assessed) were evaluated by doctors and veterinarians before the game. In fact, the subsequent measurements of the blood samples taken before the game revealed that both horses and riders were healthy. Each stallion was about 3–4 years old and weighed between 500–600 kg. They were all raised, fed and trained under the same conditions. The project involves 40 healthy male riders, aged 20–25 and weighing between 70–80 kg, who engage with these horses during games and training sessions.

### 2.1 Blood Sampling Procedure

Blood samples were taken from ten stallions and riders on each team, both half an hour before the game and within 30 min of its conclusion. Samples were drawn from the horses’ jugular vein into 10 mL tubes for serum separation. Concurrently, blood was also collected into 5 mL EDTA tubes from these horses for haematological analysis. Overall, blood was drawn from 40 horses in total. We collected venous blood samples from the riders into 10 mL tubes and extracted their sera. We also gathered simultaneous blood samples from the horses and riders into 5 mL tubes containing EDTA for haematological analysis. Overall, we took blood samples from 80 horses and 80 riders.

The serum samples were preserved at ‐80°C in a deep freezer until tested. The EDTA blood samples were analysed within 2 h of collection.

Blood samples were taken from horses and riders before and after the game to analyse cortisol, ACTH, beta‐endorphin, epinephrine, norepinephrine, T3 and T4 levels.

The haematological tests conducted included measurements of parameters such as white blood cell, red blood cell (RBC), haemoglobin (HGB), haematocrit (HCT), mean corpuscular volume, mean corpuscular haemoglobin (MCH), mean corpuscular haemoglobin concentration (MCHC), mean platelet volume, platelet distribution width (PDW), plateletcrit (PCT), red cell distribution width (RDW), red cell distribution width—coefficient of variation (RDW‐CV), red cell distribution width—standard deviation (RDW‐SD), platelet large cell ratio (P‐LCR), mid‐range cells (MID), lymphocytes (LYM), granulocytes (GRA) and platelet count (PLT).

Biochemical parameters for serum were measured, including sodium (Na), potassium (K), chloride (Cl), creatinine (CREA), urea, total protein, albumin, magnesium (Mg), alkaline phosphatase (AP), creatine kinase (CK), alanine aminotransferase (ALT) and aspartate aminotransferase (AST).

### 2.2 Hormone Analysis

We used commercial ELISA kits specific to each species to measure hormone levels in the collected blood serum. We measured cortisol (catalog no: CEA462Ge, USCN Life Science Inc., Wuhan, China), ACTH (catalogue no: CSB‐E18039Hs, CUBIO Innovation Center, Houston, TX, USA), beta‐endorphin (catalogue no: LS‐F25041, LSBio, Shirley, MA, USA), epinephrine (catalogue no: CEA858Ge, USCN Life Science Inc., Wuhan, China), norepinephrine (catalogue no: CEA907Ge, USCN Life Science Inc., Wuhan, China), T3 (catalogue no: CEA453Ge, USCN Life Science Inc., Wuhan, China) and T4 (catalogue no: CEA452Ge, USCN Life Science Inc., Wuhan, China). We followed the ELISA test application steps outlined in the manufacturer's guide. After completing the process, we determined serum hormone levels by reading the plates at 450 nm with an ELISA reader (Bio‐Tek Instruments, USA) (Can‐Şahna and Rişvanlı 2015).

### 2.3 Biochemical Analysis

Biochemical parameters, aside from electrolytes, were analysed using an automated chemistry analyser. An electrolyte analysis device determined the electrolyte levels (Safak et al., 2022).

### 2.4 Haematological Analysis

Measurements for CBC and WCDC were taken using an automatic laser haematology analyser. Blood tests were conducted at the xxxxx Central Laboratory, at the Veterinary Faculty Laboratories, or in private laboratories via service procurement.

Subsequently, the data obtained from horses before and after the game were statistically analysed based on winning and losing situations.

### 2.5 Statistical Analysis

After the study concluded, all data on animal parameters was moved into the SPSS for Windows 26 software (IBM). The data's normal distribution was verified both statistically and graphically. The Shapiro‐Wilks test was used for this. Since it did not demonstrate a normal distribution or fulfil parametric test assumptions, nonparametric tests were used–the Mann‐Whitney U test for independent groups and the Wilcoxon test for dependent groups. The Spearman correlation analysis was used to test the correlations between the variables of the horse and the rider. The threshold for statistical significance was P < 0.05.

Intra‐assay and inter‐assay variability of all these assays in riders were < 5% and < 15%, respectively and assay sensitivities for cortisol, ACTH, beta‐endorphin, epinephrine, norepinephrine, T3 and T4 were 5.15 (pg/mL), 5.25 (pg/mL), 4.53 (pg/mL), 9.74 (pg/mL), 0.112 (ng/mL), 51.4 (pg/mL) and 1.29 (ng/mL) respectively, according to the manufacturer's instructions.

Intra‐assay and inter‐assay variability of all these assays in horses were < 5% and < 15%, respectively, and assay sensitivities for cortisol, ACTH, beta‐endorphin, epinephrine, norepinephrine, T3 and T4 were 5.15 (pg/mL), 10 (ng/mL), 5.03 (pg/mL), 9.74 (pg/mL), 23.5 (pg/mL), 51.4 (pg/mL) and 1.29 (ng/mL) respectively, according to the manufacturer's instructions.

## 3 Results

Table 1 summarises the relationships between the haematological parameters of horses and riders on the victorious teams just before the competition. A clear positive correlation was seen between RDW‐CV and HGB (0.527, p < 0.05), LYM% and MID (0.654, p < 0.01), LYM% and MID% (0.532, p < 0.05), LYM% and MCHC (0.654, p < 0.01), RBC and RDVSD (0.525, p < 0.05), MDV and PDW% (0.616, p < 0.05), P‐LCR % and RDV‐CV (0.626, p < 0.05). Conversely, a negative correlation was noted between MPV and MID (‐0.609, p < 0.05), P‐LCR% and MID (‐0.624, p < 0.05), WBC and MCV (‐0.665, p < 0.01), GRA and MCV (‐0.700, p < 0.01), GRA% and MID (‐0.650, p < 0.01) GRA% and MCHC (‐0.625, p < 0.01) and MCHC and LYM (‐0.499, p < 0.01).

**TABLE 1:** Correlations between haematological parameters of horses and riders from winning teams before the game. Spearman's rho.

Horse (n = 20)	Rider (n = 20)	LYM	MID	MID%	HGB	MCHC	MCV	RDW‐CV	RDVSD	PDW%
**WBC**		—	—	—	—	—	−0.665^**^	—	—	—
**GRA**		—	—	—	—	—	−0.700^**^	—	—	—
**LYM%**		—	0.654^**^	0.532^*^	—	0.504^*^	—	—	—	—
**GRA%**		—	−0.650^**^	—	—	−0.624^**^	—	—	—	—
**RBC**		—	—	—	—	—	—	—	0.525^*^	—
**MCHC**		−0.499^*^	—	—	—	—	—	—	—	—
**RDW‐CV**		—	—	—	0.527^*^	—	—	—	—	—
**MPV**		—	−0.609^*^	—	—	—	—	—	—	0.616^*^
**P‐LCR %**		—	−0.624^*^	—	—	—	—	0.626^*^	—	—

‐ No correlation *Correlation is significant at the 0.05 level (2‐tailed). **Correlation is significant at the 0.01 level (2‐tailed). Only parameters with correlation was given.

Table 2 presents the correlation between the haematological parameters of horses and riders from victorious teams’ post‐game. Positive correlations were identified between WBC and RDW‐CV (0.573, p < 0.05), LYM and RBC (0.516, p < 0.05), LYM and RDW‐CV (0.526, p < 0.05), LYM and HCT% (0.565, p < 0.05), MID and RDW‐CV (0.483, p < 0.05), LYM% and HGB (0.610, p < 0.05), MCV and RV‐SD (0.826, p < 0.05), MCHC and MCV (0.516, p < 0.05), MCH and MCV (0.985, p < 0.01). Conversely, negative correlations were found between GRA% and HGB (‐0.586, p < 0.05), GRA% and MID (‐0.529, p < 0.05), PDW% and HGB (‐0.732, p < 0.05), PDW% and MCV (‐0.638, p < 0.05), PDW% and RDW‐SD (‐0.863, p < 0.01), PDW% and RDWCV (‐0.653, p < 0.05).

**TABLE 2:** Correlations between haematological parameters of horses and riders from winning teams after the game. Spearman's rho.

Horse (n = 20)	Rider (n = 20)	RBC	HGB	MCV	RDVCV	RDVSD	HCT%	MID%	MCV
**WBC**		—	—	—	0.573^*^	—	—	—	—
**LYM**		0.516^*^	—	—	0.526^*^	—	0.565^*^	—	—
**MID**		—	—	—	0.483^*^	—	—	—	—
**LYM%**		—	0.610^**^	—	—	—	—	—	—
**GRA%**		—	−0.586^*^	—	—	—	—	−0.529^*^	—
**MCHC**		—	—	—	—	—	—	—	0.516^*^
**MCH**		—	—	—	—	—	—	—	0.985^**^
**RDW‐SD**		—	—	—	—	—	—	—	0.826^**^
**PDW%**		—	−0.732^*^	−0.638^*^	−0.653^*^	−0.863^**^	—	—	—

‐ No correlation *Correlation is significant at the 0.05 level (2‐tailed). **Correlation is significant at the 0.01 level (2‐tailed). Only parameters with correlation was given.

Table 3 presents the correlations between the biochemical parameters of horse and rider pairs in victorious teams’ pre‐game. It reveals a negative correlation between CREA and K (‐0.559, p < 0.05), as well as Cl and K (‐0.575, p < 0.01). Positive correlations were found between AST and TP (0.504, p < 0.05), AP and TP (0.517, p < 0.05), AP and K (0.524, p < 0.05), Mg and AST (0.547, p < 0.05) and finally, Mg and AP (0.708, p < 0.01).

**TABLE 3:** Correlations between biochemical parameters of horses and riders from winning teams before the game. Spearman's rho.

Horse (n = 20)	Rider (n = 20)	TP	AST	AP	Na	K
**AST**		0.504^*^	—	—	—	—
**AP**		0.517^*^	—	—	—	0.524^*^
**CREA**		—	—	—	—	−0.559^*^
**Na**		—	—	—	0.635^**^	—
**Mg**		—	0.547^*^	0.708^**^	—	—
**Cl**		—	—	—	—	−0.575^*^

‐ No correlation *Correlation is significant at the 0.05 level (2‐tailed). **Correlation is significant at the 0.01 level (2‐tailed). Only parameters with correlation was given.

Table 4 presents a summary of the correlations between the biochemical parameters of horses and riders on victorious teams’ post‐game. Specifically, there was a negative correlation between TP and AST (‐0.627, p < 0.05). Positive correlations were noted between ALT and AP (0.650, p < 0.01), CREA and AST (0.737, p < 0.01), as well as Mg and AST (0.614, p < 0.05).

**TABLE 4:** Correlations between biochemical parameters of horses and riders from winning teams after the game. Spearman's rho.

Horse (n = 20)	Rider (n = 20)	AST	AP
**TP**		−0.627^*^	—
**ALT**		—	0.650^**^
**CREA**		0.737^**^	—
**Mg**		0.614^*^	—

‐ No correlation *Correlation is significant at the 0.05 level (2‐tailed). **Correlation is significant at the 0.01 level (2‐tailed). Only parameters with correlation was given.

None of the hormonal parameters showed any positive or negative correlation between the horses and riders from victorious teams’ post‐game.

When the winning teams examined the correlations between horses and riders from the winning teams before the game, there was a negative correlation between ACTH and T4 (‐0.529, p < 0.05).

Table 5 presents the correlation between the hormonal parameters of horses and riders from the losing teams prior to the game. There were positive correlations between cortisol and beta‐endorphin (0.569, p < 0.05), epinephrine and cortisol (0.527, p < 0.05), T3 and norepinephrine (0.486, p < 0.05), as well as T3 and T4 (0.527, p < 0.05). Conversely, negative correlations were observed between cortisol and epinephrine (‐0.519, p < 0.05), cortisol and T3 (‐0.509, p < 0.05), norepinephrine and epinephrine (‐0.488, p < 0.05) and.

**TABLE 5:** Correlations between hormonal parameters of horses and riders from losing teams before the game. Spearman's rho.

Horse (n = 20)	Rider (n = 20)	Cortisol	Beta‐endorphin	Epinephrine	Norepinephrine	ACTH	T3	T4
**Cortisol**		—	0.569^*^	−0.519^*^	—	—	−0.509^*^	—
**Epinephrine**		0.527^*^	—	—	—	—	—	—
**Norepinephrine**		0.482^*^	—	−0.488^*^	—	—	—	—
**ACTH**		—	—	—	—	−0.567^*^	—	—
**T3**		−0.567^*^	—	—	0.486^*^	—	—	0.527^*^

‐ No correlation *Correlation is significant at the 0.05 level (2‐tailed). Only parameters with correlation ware given.

None of the hormonal parameters showed any positive or negative correlation between the horses and riders from losing teams’ post‐game.

## 4 Discussion

The traditional Central Asian equestrian team game, Kök‐Börü, is relatively unknown globally. Its recognition and preservation for future generations have been hindered by the lack of extensive research outside of historical and sociological studies. The crucial role of the horse‐rider relationship in equestrian sports is universally recognised, but the physiological aspects of this relationship, especially in team sports, remain largely unexplored. Additionally, literature examining the link between physiological parameters and winning or losing in these sports is scant. The data yielded from this study offers preliminary insights into this topic.

Establishing an effective partnership between rider and horse is the key to success in equestrian sports; it lowers the risk of injury and enhances performance. Little is known about how the rider's emotional state influences the horse, but research suggests irritability affects the animal's physiological response (Merkies et al. 2014). The personality of both rider and horse can also impact their collaboration (Visser et al. 2008). In a study, Kaiser et al. (2006) found no heightened stress in horses ridden by individuals with physical or psychological disorders compared to recreational riders. Fazio et al. (2013) also found no significant hormonal response difference between horses used for therapeutic riding for physically challenged children and horses used for recreational purposes. An agitated rider can also stir emotional responses in the horse. In show jumping, connections were found between the saliva cortisol levels of the horses and their riders (Strzelec et al., 2013). Risvanli et al. (2025) reports that in Kök‐Börü games, post‐game, the riders from the winning teams showed an increase in the serum levels of WBC, GRA, GRA%, MCV, PLT, PCT, CK, ALB, AST, CREA, cortisol and ACTH compared to their levels before the game. Again in the same study, they determined that the riders' post‐game serum levels of LYM, epinephrine, norepinephrine, and T3 were lower and when pre‐game parameters of winning and losing teams were compared, it was observed that Na and T4 levels were higher in the winners. However, there is still no data on the effect of wins and losses in team equestrian sports on the riders’ and horses’ haematological, biochemical and hormonal parameters. In the presented study, when the correlations between the haematological parameters of the winners' teams' horses and riders were examined, a positive correlation was found between RDW‐CV and HGB, LYM% and MID%, LYM% and MID, LYM% and MCHC and GRA% and RDW‐CV. On the other hand, negative correlations were observed between MPV and MID, P‐LCR and MID, WBC and MCM, GRA and MCM and GRA% and both MID and MCHC. Similarly, when analysing the correlations between the post‐game haematological parameters of the winners' teams' horses and riders, positive correlations were identified between WBC and RDW‐CV, LYM and RBC, LYM and RDW‐CV, LYM and HCT%, MID and RDW‐CV, MID and MID%, LYM% and HGB, and MCV and RV‐SD. Negative correlations were found between GRA% and HGB, GRA% and MID, PDW% and HGB, PDW% and MCV, as well as PDW% and RDW‐SD. Examining the correlations between the winners' teams' pre‐game biochemical parameters of horses and riders, a negative correlation was found between CREA and K and Cl and K. Positive correlations were observed between AST and TP, AP and TP, AP and K, Mg and AST, as well as Mg and AP. In terms of post‐game biochemical parameters, a negative correlation was observed between TP and AST, while positive correlations were found between ALT and AP, CREA and AST and Mg and AST. Analysing the correlations between the pre‐game hormonal parameters of horses and riders in the winning teams, a negative correlation was found between ACTH and T4. However, no positive correlation was identified between any hormonal parameters. When investigating the correlations between the post‐game hormonal parameters of horses and riders in the winning teams, no positive or negative correlations were found for any hormonal parameter. On the other hand, in the losing teams, examining the correlations between the pre‐game hormonal parameters of horses and riders revealed positive correlations between epinephrine and norepinephrine, cortisol and beta‐endorphin, T3 and norepinephrine, norepinephrine and cortisol, as well as positive correlations between T3 and T4. Negative correlations were observed between cortisol and epinephrine, as well as cortisol and T3. However, when analysing the correlations between the post‐game hormonal parameters of horses and riders in the losing teams, no positive or negative correlations were identified for any hormonal parameter.

This study provides new insight into the physiological responses of riders and horses to the demands of traditional Central Asian equestrian team sport, Kök‐Börü. Although the results illustrate strong relationships between a range of haematological and biochemical and endocrine parameters, they should be considered within the confines of athletic performance, response to stress and horse‐rider interactions.

As the positive correlations observed for the LYM% of the horses and the winners' riders correlate positively with the MCHC or GRA% and RDW‐CV, linking immune function with oxygen‐carrying capacity, it is interesting that the LYM% of the horses from the winning teams was high. On the other hand, negative correlations such as that found between GRA% and MCHC may demonstrate greater physiologic responses to exertion in horses versus riders. These results, along with the positive correlation between WBC and RDW‐CV among individuals, suggest that immune activation may also be a common response to the physical stressors associated with the game of Kök‐Börü in both groups. It is not clear, though, whether these shifts are primarily due to competition‐induced stress, physical activity.

In terms of biochemical responses, the positive correlations between ALT and AP, CREA and AST and Mg and AST in post‐game samples of winning teams suggest metabolic and muscular adaptations following high‐intensity activity. The significant negative correlation between TP and AST may point to shifts in protein metabolism due to exertion. The differences observed pre‐ and post‐game highlight the physiological demands placed on both horses and riders, yet the specific role of winning or losing in these biochemical changes requires further exploration. The presence of correlations between CREA and K and Cl and K pre‐game, could indicate baseline metabolic differences that influence performance.

The hormonal correlations in losing teams, such as between cortisol and beta‐endorphin or norepinephrine and cortisol, suggest a heightened stress response compared to winners. The inverse relationships observed between cortisol and epinephrine or cortisol and T3 further support the notion of varied physiological responses to stress and performance outcomes. The absence of significant post‐game hormonal correlations in both winners and losers raises questions regarding recovery mechanisms and stress adaptation in equestrian athletes. These findings align with previous research on stress in equestrian sports but warrant further investigation with more controlled study designs.

## 5 Conclusions

This study serves as an initial exploration into the physiological relationship between horses and riders in Kök‐Börü. The identified correlations indicate intricate biochemical and haematological interactions that are affected by factors such as exertion, competition results, and potentially stress. Future research should build on these findings using structured methodologies that consider training effects, physical conditioning, and varying stress responses in equestrian team sports. By deepening our understanding of these interactions, we can help preserve and promote traditional equestrian sports while improving the welfare of both athletes and animals.

## Author Contributions

**Ali Rişvanlı, İsmail Şen, Kanat Canuzakov**: Conceptualisation; data curation; formal analysis; investigation; methodology; project administration; resources; software; validation; visualization; writing – original draft; writing – review and editing. **Askarbek Tülöbayev, Abuzer Taş, Nezahat Ceylan, Ünal Türkçapar, Ulanbek Alimov**: Conceptualisation; funding acquisition; investigation; methodology; supervision; writing – review and editing. **Arina Kazakbayeva, Ayday Cunuşova, Nur Abdımnap Uulu**: Investigation; methodology; supervision. **Burak Fatih Yuksel, Mert Turanli, Muhammed Uz, Metin Bayraktar, Nuriddin Ruzikulov**: Investigation; methodology; resources.

## Ethics Statement

The project has been approved by both the Animal Ethics Committee at Manas University in Kyrgyzstan‐Turkey (Document Date and Number: 20.01.2023‐2023/01) and the Human Experiments Ethics Committee at the Ministry of Health in Kyrgyzstan (Document Date and Number: 15.01.2023‐2023/03).

## Conflicts of Interest

The authors declare no conflicts of interest.

### Peer Review

The peer review history for this article is available at https://www.webofscience.com/api/gateway/wos/peer‐review/10.1002/vms3.70356.

## Data Availability

The data that support the findings of this study are available from the corresponding author upon reasonable request.
